# Broad-Spectrum Supramolecularly Reloadable Antimicrobial Coatings

**DOI:** 10.1021/acsami.4c04705

**Published:** 2024-06-03

**Authors:** Fiora Artusio, Lukas Müller, Nicolò Razza, Inês Cordeiro Filipe, Francesca Olgiati, Łukasz Richter, Edoardo Civera, Melis Özkan, Matteo Gasbarri, Louisa Rinaldi, Heyun Wang, Esther Garcìa, Julie Schafer, Lise Michot, Sophie Butot, Leen Baert, Sophie Zuber, Marcus Halik, Francesco Stellacci

**Affiliations:** † Institute of Materials, Ecole Polytechnique Fédérale de Lausanne (EPFL), 1015 Lausanne, Switzerland; ‡ Organic Materials & Devices, Institute of Polymer Materials, Interdisciplinary Center for Nanostructured Films (IZNF), Friedrich-Alexander-Universität Erlangen-Nürnberg, Cauerstraße 3, 91058 Erlangen, Germany; § Nestlé Research, Institute of Food Safety and Analytical Sciences, Vers-chez-les-Blanc, Box 44, 1000 Lausanne, Switzerland; ∥ Interfaculty Bioengineering Institute, Ecole Polytechnique Fédérale de Lausanne (EPFL), 1015 Lausanne, Switzerland

**Keywords:** antimicrobial surfaces, scalability, quaternary ammonium compounds, supramolecular interactions, reloading

## Abstract

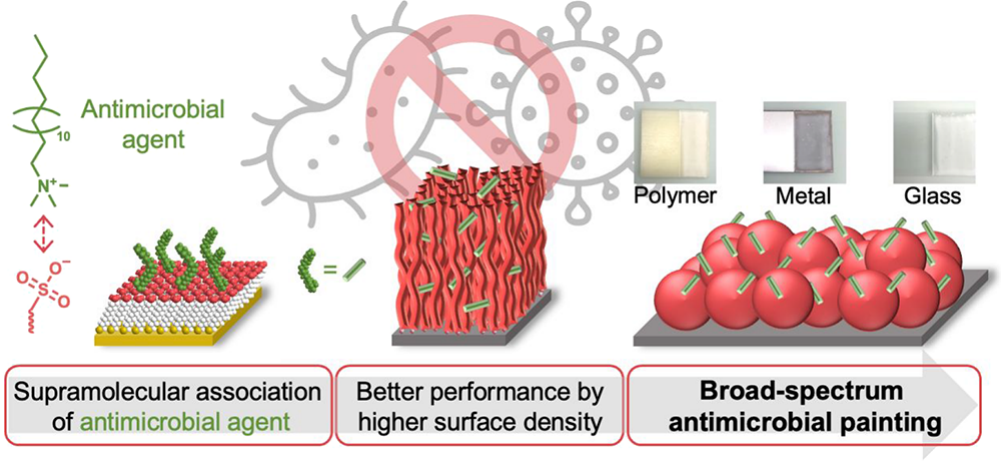

Antimicrobial surfaces limit the spread of infectious diseases. To date, there is no antimicrobial coating that has widespread use because of short-lived and limited spectrum efficacy, poor resistance to organic material, and/or cost. Here, we present a paint based on waterborne latex particles that is supramolecularly associated with quaternary ammonium compounds (QACs). The optimal supramolecular pairing was first determined by immobilizing selected ions on self-assembled monolayers exposing different groups. The QAC surface loading density was then increased by using polymer brushes. These concepts were adopted to develop inexpensive paints to be applied on many different surfaces. The paint could be employed for healthcare and food production applications. Its slow release of QAC allows for long-lasting antimicrobial action, even in the presence of organic material. Its efficacy lasts for more than 90 washes, and importantly, once lost, it can readily be restored by spraying an aqueous solution of the QAC. We mainly tested cetyltrimethylammonium as QAC as it is already used in consumer care products. Our antimicrobial paint is broad spectrum as it showed excellent antimicrobial efficiency against four bacteria and four viruses.

## Introduction

The rapid growth of the human population together with the increasing tendency toward globalization has facilitated the spread of infectious diseases all over the globe, as emblematically demonstrated by the coronavirus disease 2019 (COVID-19) pandemic. Great efforts have been made to improve the toolkit available to fight against such diseases, which counts for prophylactic treatments like vaccinations, antiviral drugs, antibiotics, regular surface disinfection, and antimicrobial surfaces.^1−4^ The use of antimicrobial surfaces is desirable in many fields, from healthcare and food production facilities to surfaces in public spaces that are often touched. For most of these applications, one would need a simple, inexpensive, and scalable solution to make very different surfaces antimicrobial.

Surface disinfection is the most common practice to guarantee hygienic conditions. It usually involves chemical disinfectants (e.g., ethanol, chlorine, surfactants), UV irradiation, or heat.^5,6^ Surfactants carrying long hydrophobic chains are well-known disinfecting agents thanks to their ability to disrupt membranes and protein assemblies in the virus/bacterium structure.^7,8^ However, surfactants can easily be washed off of surfaces after their application. Their toxicity and limited biodegradability, coupled with their massive daily use in industry and households, raise environmental concerns.^9^ Furthermore, the short lifetime of the disinfecting action limits large-scale applications as it requires regular surface treatments and high surfactant consumption, without guaranteeing a continuous antimicrobial action. The need to find efficient ways to protect surfaces is obvious. Self-disinfecting surfaces represent one possible solution to this demand.

Two broad classes of approaches exist to produce antimicrobial surfaces, adhesion-prevention or contact-deactivation.^10^ The former often involves the use of nature-inspired superhydrophobic surfaces^11^ to prevent microorganism adhesion.^12−14^ To date, these approaches are rather complex and limited to a few flat surfaces, and their durability remains unclear. There exists a plethora of contact-deactivation approaches; they all require a chemical modification of surfaces to impart specific bactericidal and virucidal actions. For example, inorganic antimicrobial agents, such as silver, gold, or copper nanoparticles, have been blended into polymeric matrixes or used to prepare graphene-based composites having antimicrobial features.^15,16^ Titanium oxides or lignin led to the photocatalytic disruption of viruses via reactive oxygen species (ROS) surface generation.^17,18^ Biocides as quaternary ammonium compounds or chlorine were immobilized on silica nanoparticles^19^ or polymers,^20,21^ which were highly effective against bacteria. In addition, antimicrobial peptides have also been conjugated with titanium and demonstrated antibacterial activity.^22^ Polymeric materials with inherently antimicrobial properties have also been designed based on charged multiblock polymers,^23^*N*,*N*-dodecyl,methyl-polyethylenimines,^24^ and oxazolines.^25^ Plasma processing has also been proposed to impart antiviral properties to polymers by modifying surface morphology and/or chemistry.^26^ Yet, none of these approaches have reached widespread application on the market. Common shortcomings are the loss of antimicrobial activity when the release of the active compounds stops, either because of depletion or because organic contaminations form an unsurmountable barrier. In such cases, effective solutions have not been found, as efficient reloading has not been reported, and most surfaces lose their antimicrobial properties when repeatedly washed.^23,27^

Here, we present a method for the preparation of reloadable broad-spectrum antimicrobial coatings based on a paint that can be applied to polymers, metals, and glass surfaces. The antimicrobial action of the coating withstood repeated washing, organic contamination, or surface aging. The coating exerted a broad-spectrum antimicrobial action against four model viruses (Herpes Simplex Virus Type 2, Influenza virus, Severe Acute Respiratory Syndrome Coronavirus 2, and Pseudomonas phage phi6) and four model bacteria (*Staphylococcus aureus*, *Listeria monocytogenes*, *Escherichia coli*, and *Salmonella enteritidis*). A paint based on latex particles exposing sulfonate groups, after drying on a surface, is loaded with cetyltrimethylammonium (CTA+) ions. The use of supramolecular electrostatic interactions to immobilize CTA+ allows for the reloading of the coating once the antimicrobial action is lost, i.e., after 90 washes for *S. aureus*. The surface/active molecule combination was determined by selecting two model surfactant ions commonly used in industry,^28^ one positively charged (CTA+) and one negatively charged, dodecylsulfate (DS-). The ions were immobilized on self-assembled monolayers (SAMs) exposing different groups. The coordination of CTA+ to sulfonate groups immobilized on the surface led to the highest reduction in the Herpes Simplex Virus Type 2 (HSV-2) titer. A comparison in antiviral efficacy between CTA+-loaded SAMs and polymer brushes of varying thickness allowed us to determine that the surface loading density is the key to achieving very high efficiency. These results led us to design a strategy based on a paint that can be readily loaded to a high degree while at the same time being inexpensive, scalable, and easily applicable on many surfaces. The efficacy of CTA+ was compared to that of other quaternary ammonium compounds (QACs) and found to be one of the best-performing molecules, yet the main driver for the choice of CTA+ is that it is a Generally Regarded As Safe (GRAS) molecule that is already approved for use in personal care products. In addition, CTA+ has nonhemolytic properties,^29^ expanding the potential of our approach also to future medical applications.

## Results

To develop broad-spectrum reloadable antimicrobial surfaces based on the use of supramolecular interactions to slowly release and then allow for the reloading of antimicrobial molecules, we started by determining the best surface group/immobilized molecule charge pairing.

**Figure 1:**
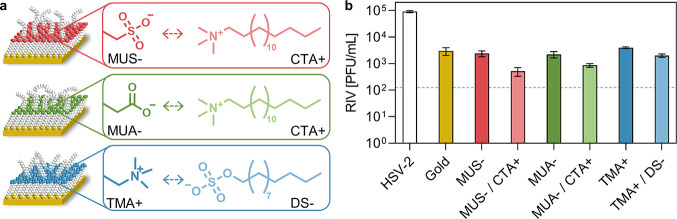
(a) Schematic drawing of the supramolecular interactions between MUS- (top), MUA- (middle), and TMA+ (bottom) SAMs and antimicrobial CTA+/DS-. (b) Antiviral test against HSV-2 performed on pristine SAMs and SAMs loaded with surfactants (75 min inoculation). HSV-2 inoculum was 9 × 10^4^ PFU/mL. RIV stands for recovered infectious virus, as quantified by a plaque assay. The limit of detection was 125 PFU/mL, as shown by the dashed line. White bar refers to HSV-2 inoculum, yellow bar to gold controls, red bars to recovered virus on MUS- SAMs (without and with CTA+), green bars to recovered virus on MUA- SAMs (without and with CTA+), and blue bars to recovered virus on TMA+ SAMs (without and with DS-).

SAMs were selected as initial test surface-coating because, in our opinion, they are the simplest platform to assess the optimal combination of surface-exposed groups and surfactant chemistry since they provide controlled and reproducible surface functionalization.^30,31^ SAMs on gold surfaces were formed using thiolated alkanes either ω-terminated with sulfonate (sodium 11-mercapto-1-undecanesulfonate, MUS-) or carboxylate (11-mercaptoundecanoic acid, MUA-) groups to be coupled to CTA+^32−34^ or ω-terminated with ammonium (11-mercaptoundecyl)-N,N,N-trimethylammonium bromide, TMA+) groups to be coupled with DS-. Dose–response assays of the two active compounds are reported in Figure S1. A scheme of the investigated surfaces and the chemical structure of the immobilized active compounds are reported in Figure 1a. SAMs, either as-prepared or surfactant-loaded, were characterized in terms of contact angle and thickness, as reported in SI (Table S1). An increase in thickness was observed for all the SAMs upon loading with the surfactant, suggesting successful surfactant immobilization. X-ray photoelectron spectroscopy (XPS) confirmed the presence of characteristic chemical elements of the SAMs and surfactants [high-resolution (HR) XPS spectra of N 1s/S 2s regions are shown in Figure S2]. The SAMs were investigated in terms of antiviral activity against a model virus, HSV-2. The results of the antiviral tests are reported in Figure 1b. SAMs loaded with either active compound showed higher virus deactivation compared to that of the corresponding as-prepared SAM, as expected. The deactivation was higher when CTA+ was used, independently of the chemical group selected for its immobilization.

**Figure 2:**
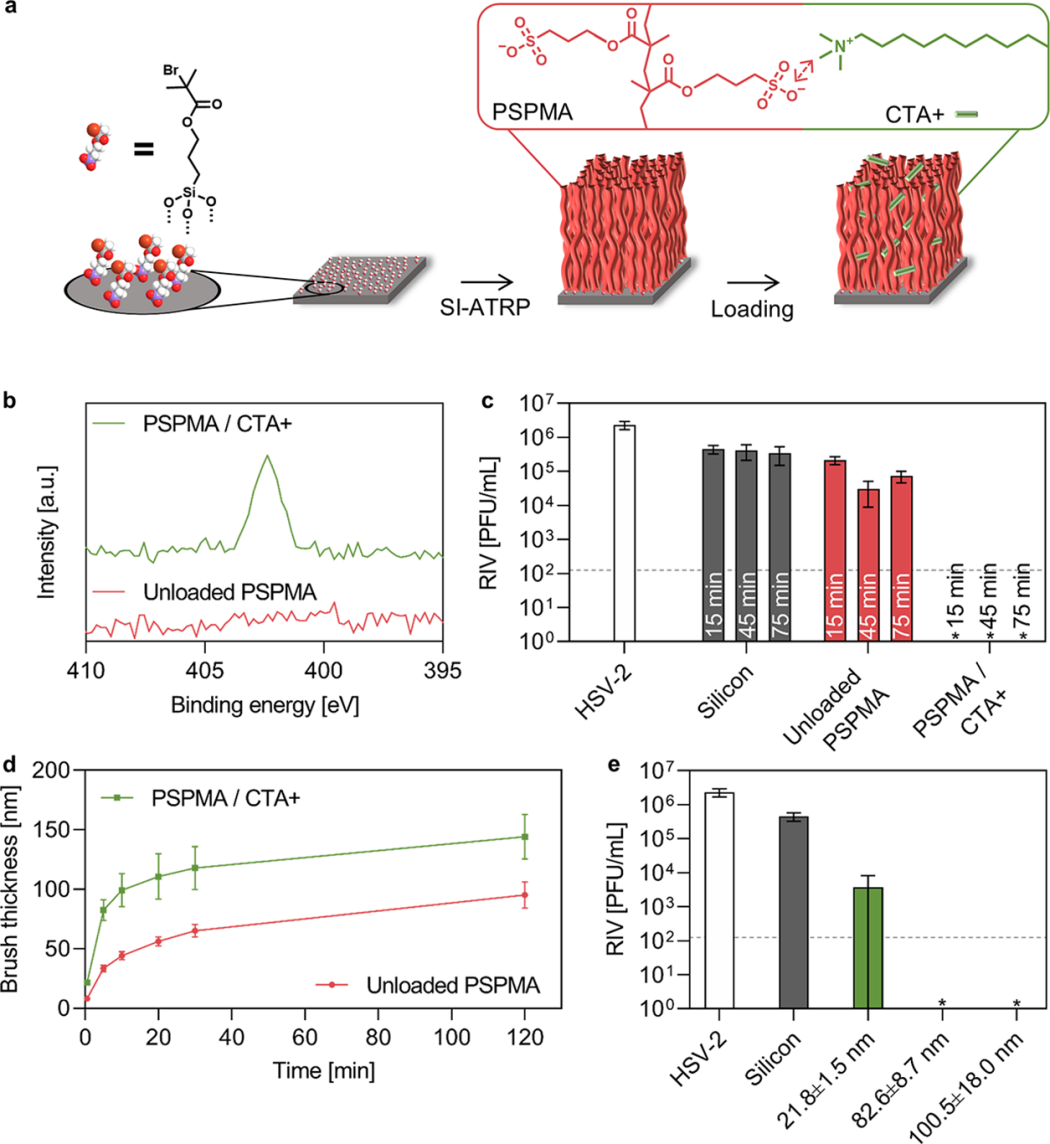
(a) Schematic drawing of SI-ATRP of PSPMA polymer brushes and their loading with CTA+. (b) HR-XPS of the N 1s region of unloaded and CTA+-loaded PSPMA brushes. (c) Antiviral test against HSV-2 performed on unloaded and CTA+-loaded PSPMA brushes at various inoculation times (15, 45, and 75 min). The virus inoculum was dried off the surface. (d) Unloaded and CTA+-loaded brush thickness as a function of ATRP time, as measured by ellipsometry. (e) Antiviral test against HSV-2 performed on brushes with different thicknesses (15 min inoculation). The antiviral action of the CTA+-loaded brushes was related to their thickness. HSV-2 inoculum was 2 × 10^6^ PFU/mL. RIV stands for recovered infectious virus, as quantified by the plaque assay. The limit of detection was 125 PFU/mL, as shown by the dashed line. White bars refer to HSV-2 inoculums, grey bars to silicon controls, red bars to recovered virus on as-prepared brushes, and green bars to recovered virus on CTA+-loaded brushes. *stands for no observed infection.

The modest antiviral action (<1 log_10_) exerted by SAMs in reference to gold was attributed to the limited loading capacity of active compounds. To test this hypothesis, we adopted an alternative surface design based on polymer brushes that allowed us to achieve significantly higher loading as well as to vary the loading by controlling the brush thickness. We chose polysulfopropyl methacrylate (PSPMA) as a brush with sulfonate groups as the coordinating moiety because of the lower apparent p*K*_a_ value and hence the presence in the deprotonated state in a wider pH window compared to carboxylate groups.^35^ Brushes were prepared by surface-initiated atom transfer radical polymerization (SI-ATRP) after immobilization of the initiator on a silicon wafer, as shown in Figure 2a. PSPMA brushes were loaded with CTA+, as confirmed by HR-XPS on the N 1s region (Figure 2b), and tested against HSV-2. The antiviral activity of the brush was tested considering different virus inoculation scenarios, referred to in this article as wet-to-dry and wet-to-wet (see SI for detailed protocols). As outlined in Figure S3, when the evaporation of the virus inoculum was prevented, a 4 log_10_ (99.99%) reduction in the virus titer was observed. However, allowing the virus inoculum to dry on the surface has been recently suggested to better represent real-life scenarios^27^ and was therefore selected for the following part of this study. Multiple time points (15, 45, and 75 min starting from the inoculation time) were selected to test the antiviral activity of the brushes when an HSV-2 inoculum was dried. The results reported in Figure 2c indicate rapid (15 min) and more than 4 log_10_ (99.99%) virus deactivation, independent of the method used to test the antiviral action of the surface. These results also suggested the enhanced performance of the brush molecular architecture in comparison to SAMs, which was attributed to the higher surface density of sulfonate groups.

To further study this aspect, we prepared PSPMA brushes with different thicknesses. As sketched in Figure 2d, the brush thickness can be tuned by acting on the polymerization time. A thickness range between 8 and 95 nm for the as-prepared PSPMA brushes was obtained by stopping ATRP at various time points ranging from 30 s up to 2 h. The thickness differences between as-prepared and CTA+-loaded samples were associated with swelling effects by incorporation of the surfactant into the brush architecture, which is a previously reported effect.^36^ As reported in Figure 2e, the reduction in the HSV-2 viral titer was dependent on the brush thickness and hence on the number of sulfonate groups. When the brush thickness was above 80 nm, more than 4 log_10_ (99.99%) virus deactivation was achieved, whereas slightly more than 2 log_10_ (99%) titer reduction was observed for approximately 20 nm thick brushes. A high surface density of sulfonate groups is therefore crucial for achieving high antiviral activity since the number of sulfonate groups per unit area increases from approximately 5 groups/nm^2^ for MUS SAMs^37^ to approximately 50 and 200 groups/nm^2^ for 20 and 80 nm thick PSPMA brushes,^38^ respectively.

To further challenge the robustness of our supramolecular approach, we also tested the antiviral activity of the brushes in the presence of organic contamination and against a second virus. As reported in Figure S4, PSPMA brushes reduced the HSV-2 titer by more than 4 log_10_ (99.99%) even after being contaminated with finger grease (and optionally cleaned afterwards) 5, 15, and 30 times. Furthermore, PSPMA brushes could reduce the SARS-CoV-2 titer by more than 3 log_10_ (99.9%) after 75 min from inoculation (Figure S5).

**Figure 3:**
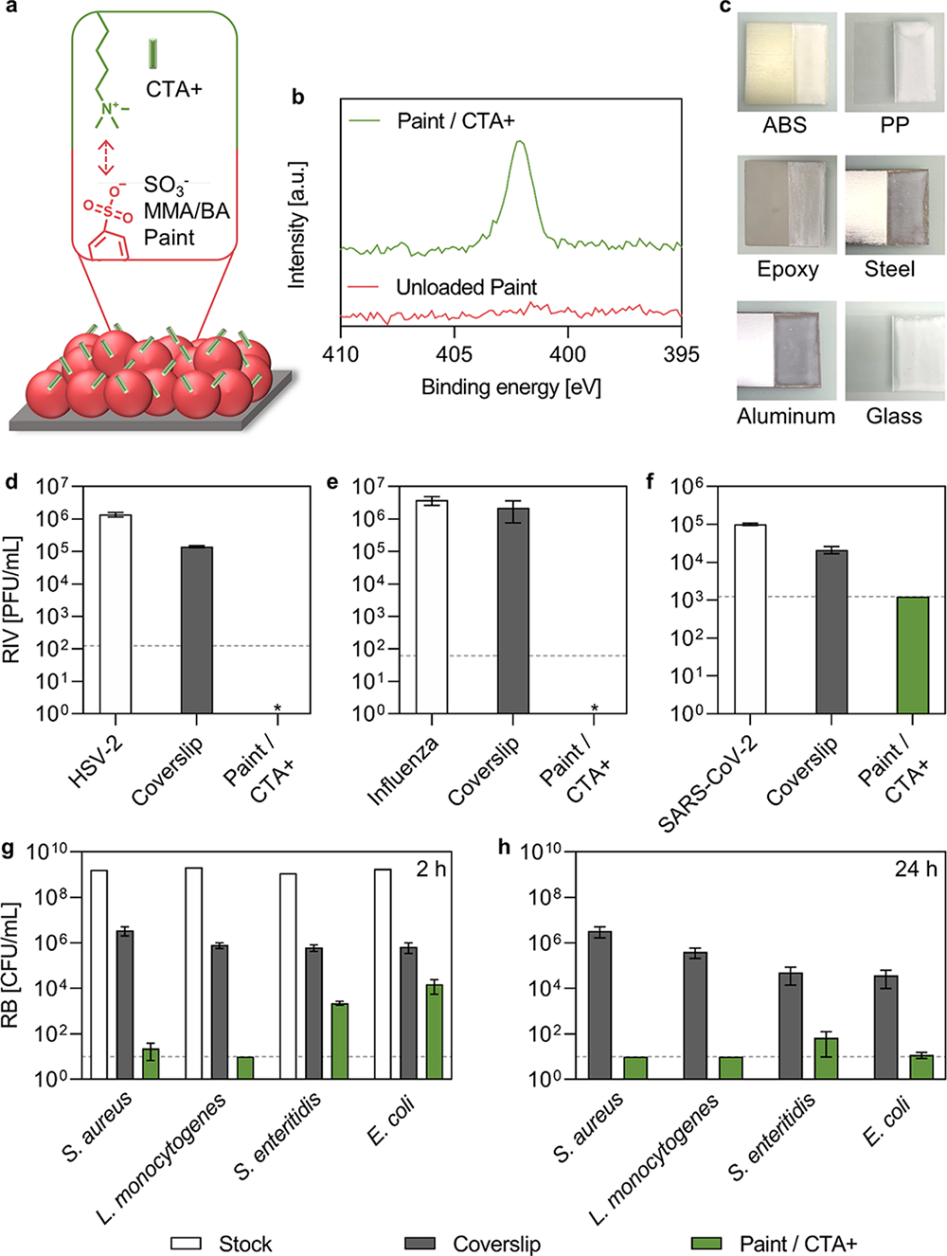
(a) Schematic drawing of the particle-based paint interacting with CTA+ ions. (b) HR-XPS of the N 1s region of unloaded and CTA+-loaded painted surfaces. (c) Coating applied to different materials: acrylonitrile butadiene styrene (ABS) plastic, polypropylene (PP), epoxy, stainless steel, aluminum, and glass. Antiviral tests performed on painted surfaces loaded with 1 mM CTA+ against (d) HSV-2 (inoculum = 1 × 10^6^ PFU/mL, inoculation time = 30 min), (e) Influenza A (inoculum = 4 × 10^6^ PFU/mL, inoculation time = 30 min), (f) SARS-CoV-2 (inoculum = 1 × 10^5^ PFU/mL, inoculation time = 2 h). RIV stands for recovered infectious virus, as quantified by a plaque assay. The limits of detection were 125, 62, and 1250 PFU/mL, respectively, as shown by the dashed line. Antibacterial tests performed on painted surfaces loaded with 1 mM CTA+ against *S. aureus* (inoculum = 2 × 10^9^ CFU/mL), *L. monocytogenes* (inoculum = 2 × 10^9^ CFU/mL), *S. enteritidis* (inoculum = 1 × 10^9^ CFU/mL), and *E. coli* (inoculum = 2 × 10^9^ CFU/mL), after (g) 2 and (h) 24 h. RB stands for recovered bacteria. The limit of detection was 10 CFU/mL, as shown by the dashed line. White bars refer to microorganism inoculums, grey bars to surface controls, and green bars to recovered microorganisms from the CTA+-loaded surfaces. *stands for no observed infection.

The previous results obtained on SAMs and brushes showed that supramolecular interactions can be used to develop antiviral surfaces. However, their synthesis is limited to surfaces having specific chemistry (gold for SAMs, presence of −OH groups for brushes) and, especially in the case of brushes, can be quite expensive. As a further step, we developed a technological solution to prepare reloadable antimicrobial paints that can be applied to a wide variety of surfaces. The overarching idea was to create a versatile and inexpensive paint. This paint is composed of polymeric particles made of butyl acrylate (BA) and methyl methacrylate (MMA). Hereinafter, we will refer to this formulation as “paint” even if, unlike a commercial paint formulation, it does not contain dispersants, wetting agents, fillers, or pigments.^39^ A polymerizable surfactant, i.e., surfmer, was used to stabilize the formulation and avoid the leaching of surfactants.^40,41^ The surfmer was selected so as to expose sulfonate groups on the particles’ surface and to allow for the CTA+ immobilization via supramolecular interactions, as illustrated in Figure 3a. The presence of CTA+ was proved by the appearance of the N 1s peak in the corresponding region by HR-XPS, as reported in Figure 3b. Additional data including SEM micrographs, contact angle, and elemental composition determined by XPS of the CTA+-loaded coating are reported in Figures S6, S7 and Table S2, respectively. CTA+ loading was achieved by dipping the coated surface in the CTA+ solution. The dipping procedure did not lead to peeling off of the painted layer. However, when an excess of paint was used to prepare the coating, macro-cracking compromised the quality of the coating, promoting the peeling off during the dipping phase. In comparison to brushes, the concentration of the CTA+ dipping solution was increased from 0.1 to 1 mM to enhance the surface loading. As an alternative, the incorporation of CTA+ into the paint liquid formulation was also tested (see Figure S8). However, a high reduction in HSV-2 viral titer was achieved only when CTA+ was loaded by dipping, probably because of insufficient surface exposure of CTA+ when directly added to the liquid formulation. Different surfaces including polymers (acrylonitrile butadiene styrene, polypropylene, epoxy), metals (stainless steel and aluminum), and glass were successfully coated with the paint (Figure 3c). The adhesion of the paint was evaluated according to ASTM D3359 and resulted in a 5B grade for all the substrates, except for polypropylene (0B). The excellent adhesion of the paint on several substrates was attributed to the favorable interactions between the paint, which is made of a mixture of two monomers having different hydrophilicities, and the substrates during the application. A primer layer could be applied before the paint to enhance the adhesion on materials showing low adhesion grades.

The broad-spectrum antimicrobial action of painted surfaces loaded with CTA+ was tested against a variety of viruses and bacteria. As a control, a plastic coverslip was used. Surfaces were able to exert a strong and quasi-immediate antiviral action: more than 4 log_10_ reduction (>99.99%) was obtained for HSV-2 (Figure 3d) and Influenza (Figure 3e) in 30 min, and more than 1.6 log_10_ reduction (>97.5%) reduction was observed for SARS-CoV-2 (Figure 3f) in 2 h. A similar test was also carried out on Pseudomonas phage phi6, showing more than 4 log_10_ reduction (>99.99%) in 2 h (see Figure S9). Pseudomonas phage phi6 has been used as a SARS-CoV-2 surrogate,^42,43^ but the marked differences observed in our tests may suggest that this bacteriophage is not always a good model for SARS-CoV-2. A similar performance was obtained when testing model Gram-positive bacteria, as *S. aureus* (>4 log_10_ reduction) and *L. monocytogenes* (>4 log_10_ reduction) after 2 h (Figure 3g). A high antibacterial action was also observed for Gram-negative bacteria, as *E. coli* (>3 log_10_ reduction) and *S. enteritidis* (>3 log_10_ reduction) but required a longer time for deactivation (24 h, Figure 3h). Such a difference was attributed to the Gram-negative bacteria outer membrane containing lipopolysaccharides in addition to the peptidoglycan layer,^44^ which is also thicker compared to Gram-positive bacteria, which may make their deactivation more challenging.

**Figure 4:**
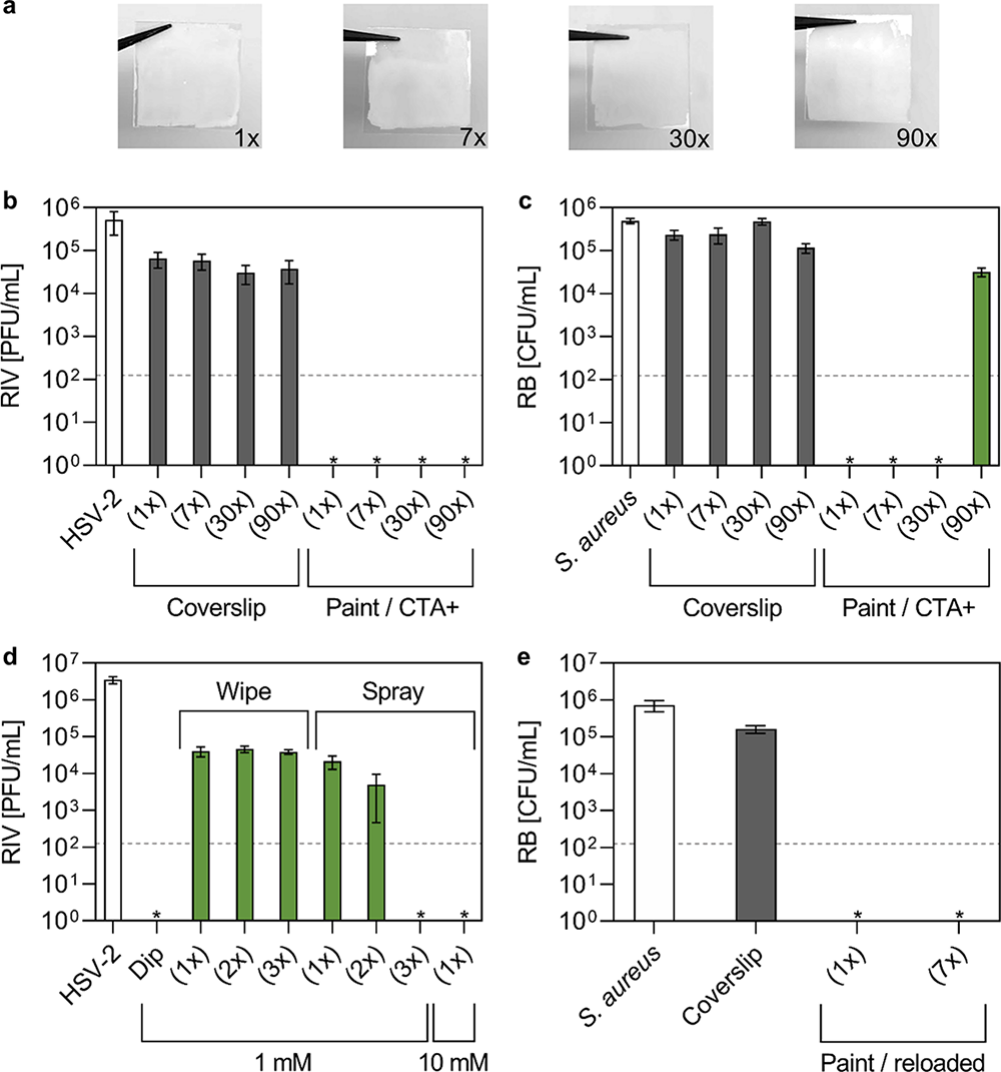
(a) Photographs of the painted surfaces loaded with CTA+ washed multiple times. (b) Antiviral (HSV-2 inoculum = 5 × 10^5^ PFU/mL, inoculation time = 30 min) and (c) antibacterial (*S. aureus* inoculum = 5 × 10^5^ CFU/mL, inoculation time = 30 min) tests performed on painted surfaces loaded with 1 mM CTA+ washed 1, 7, 30, and 90 times. (d) Antiviral tests (HSV-2 inoculum = 4 × 10^6^ PFU/mL, inoculation time = 30 min) to identify a scalable loading protocol. The coating was loaded either by dipping the surface in 1 mM CTA+ (“Dip”) or with 1, 2, or 3 passages with wipes impregnated with a 1 mM CTA+ solution or sprays of the 1 mM CTA+ solution. Also, a single application of 10 mM CTA+ was tested. (e) Antibacterial tests (*S. aureus* inoculum = 7 × 10^5^ CFU/mL, inoculation time = 30 min) after 90× washes and reloading with 1 mM CTA+. Surfaces were washed 1 and 7 times and recovered the antibacterial activity. RIV and RB stand for recovered infectious virus, as quantified by the plaque assay, and recovered bacteria, respectively. The limits of detection were 125 PFU/mL and 125 CFU/mL for the antiviral and the antibacterial tests, respectively, as shown by the dashed line. White bars refer to microorganism inoculums, grey bars to surface controls, and green bars to recovered microorganisms from the painted surfaces. *stands for no observed infection.

The stability and durability of CTA+ immobilized on the painted surfaces were assessed by washing the surfaces multiple times with water. Tests were performed washing the surface 1, 7, 30, and 90 times to simulate daily cleaning for a day, a week, one month, and three months, respectively. As shown in Figure 4a, the paint was able to withstand 90 washes without compromising its physical appearance. The antimicrobial activity of the painted surfaces was assessed after each set of washings. Figure 4b shows that the antiviral activity against HSV-2 was fully preserved, even after 90 washes. As far as *S. aureus* was concerned, the antibacterial activity was lost after 90 washes, as can be seen in Figure 4c. Nevertheless, the surfaces can easily be replenished by dipping them in a 1 mM CTA+ solution to restore their antimicrobial activity, as shown in Figure 4d. The replenished coating was even able to withstand more washing cycles (1× and 7×) without losing efficacy (Figure 4e). Alternative approaches, closer to real-life scenarios, for the loading of the active compound were also studied. Painted surfaces were successfully reloaded multiple times with wipes prewetted with 1 mM CTA+ or sprayed with 1 mM or 10 mM CTA+ solutions. Figure 4d highlights the achievement of the same reduction in the HSV-2 viral titer as the impregnation approach by spraying the surfaces three times with 1 mM CTA+ or with a single application of a 10 mM CTA+ spray. A single application of 10 mM CTA+ spray was also effective in restoring the antimicrobial activity after 90× washed surfaces (see Figure S10). Such a method represents a facile route to load and reload the painted surfaces with CTA+.

**Figure 5:**
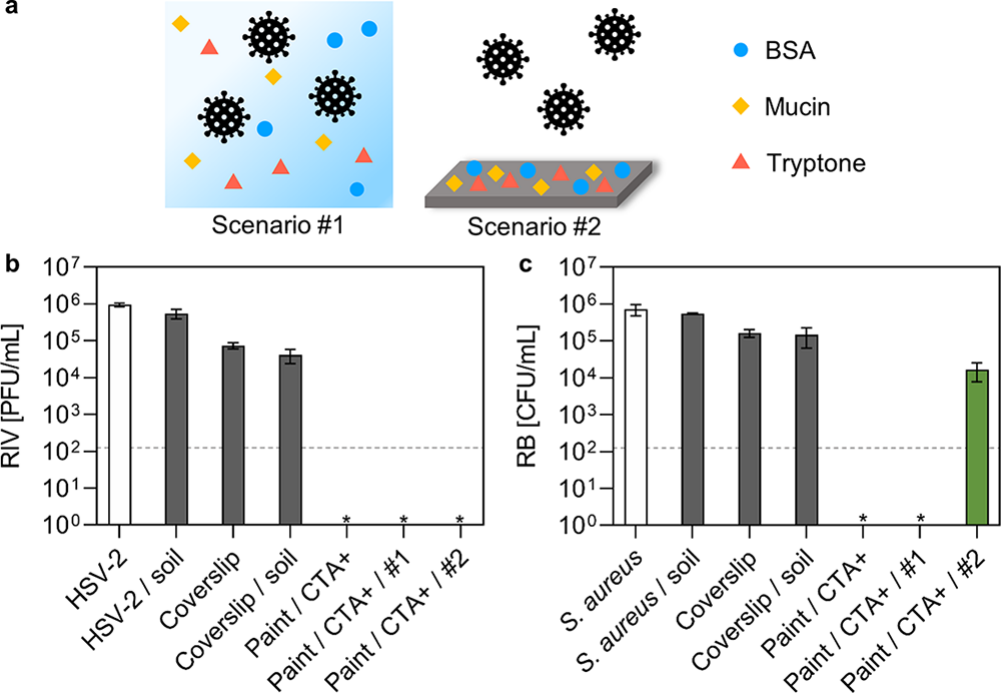
(a) Schematic drawing of the two conditions tested: scenario #1 refers to soil contamination introduced in the inoculum, whereas scenario #2 refers to soil contamination on the surfaces. (b) Antiviral test (HSV-2 inoculum = 1 × 10^6^ PFU/mL, inoculation time = 30 min) 2 and (c) antibacterial test (*S. aureus* inoculum = 7 × 10^5^ CFU/mL, inoculation time = 30 min) performed considering scenarios #1 and #2 of soil contamination. RIV and RB stand for recovered infectious virus, as quantified by the plaque assay, and recovered bacteria, respectively. The limits of detection were 125 PFU/mL and 125 CFU/mL for the antiviral and the antibacterial tests, respectively, as shown by the dashed line. White bars refer to microorganism inoculums, gray bars to surface controls, and green bars to recovered microorganisms from the painted surfaces. *stands for no observed infection.

Furthermore, the antimicrobial performance of the surfaces was assessed by considering conditions that would mimic real-life scenarios. As illustrated in Figure 5a, two different scenarios were considered. Scenario #1 foresees the introduction of “soil” contamination, i.e., a mixture of bovine serum albumin (BSA), tryptone, and mucin, into the microorganism inoculum. This would simulate the medium in which viruses and bacteria are normally present when encountering fomites, such as lung expectorate. Scenario #2 involves the contamination of surfaces with soil to simulate the condition in which surfaces normally are found in realistic scenarios, i.e., contaminated with dirt, finger grease, *etc*. In this approach, a “crust” was formed on the surfaces to be tested by evaporating the soil solution. As reported in Figure 5b, the introduction of soil contamination in the virus inoculum (HSV-2/soil) or on the surface control (Coverslip/soil) did not significantly alter the recovered infectious virus, confirming the robustness of the test. The painted surfaces were still able to reduce the HSV-2 titer by more than 3 log_10_. In the case of bacteria, some colonies were observed under scenario #2 conditions; see Figure 5c. This was attributed to the larger size of bacteria compared to viruses, possibly hindering their diffusion through the thick crust layer to get in contact with CTA+ on the surface.

To challenge the antimicrobial properties of the CTA+-loaded coating, some aging tests were also performed. Surfaces were either exposed to controlled lighting conditions (4 h under a UV lamp, 250 W) or mild heating (24 h at 40 °C). The antibacterial activity of the coating was tested, as outlined in Figure S11. The aging test did not compromise the antimicrobial activity of the coating, which was still able to lead to more than 3 log_10_ reduction against *S. aureus*. The contamination and aging tests highlight the potential of the application of this approach to real-life scenarios and high-touch surfaces.

The immobilization of CTA+ on the paint via supramolecular interactions paves the way for a flexible approach to self-disinfecting surfaces. This approach can potentially be extended to any QAC molecule. The immobilization of other QACs, having either two hydrocarbon chains or one hydrocarbon chain and an aromatic ring, was successfully obtained (see the XPS spectra in Figure S12). QACs with aromatic rings had comparable antibacterial activity to CTA+, whereas QACs with two hydrocarbon chains were less performing (see Figures S13 and S14). This feature of the coating allows us to easily change the antimicrobial molecule, which is highly desirable depending on the specific need or to fight against antimicrobial resistance.^45^

## Conclusions

Here, we developed a broad-spectrum antimicrobial paint that can be applied on various surfaces, including polymers, metals, and glass. To identify the best surface design, a workflow based on different systems was established. SAMs were used to select the best combination of surface and active compound chemistry independently from the paint architecture. The coordination of a positively charged quaternary ammonium compound, CTA+, to sulfonate groups was selected. The key role of sulfonate groups immobilized on the surface was disclosed by preparing sulfonated brushes with different thicknesses. The antiviral activity against HSV-2 depended on the brush thickness and therefore on the number of sulfonate groups. A paint carrying a high moiety density available for CTA+ coordination was designed based on waterborne latex particles. The antimicrobial action was broad-spectrum as the paint was active against HSV-2, Influenza virus, Pseudomonas phage phi6, and SARS-CoV-2, as well as on multiple Gram-positive and Gram-negative bacteria. The antimicrobial activity had a long lifetime, withstanding up to 90 washing cycles, and was maintained in the presence of organic contaminations, and after aging tests.

Overall, the robustness of supramolecular interactions for the development of antimicrobial surfaces allowed the design of broad-spectrum, reloadable, and cost-effective antiviral and antibacterial coatings. Waterborne latexes can be synthesized via industrial-like semicontinuous conditions, and the coating can easily be loaded and reloaded by spraying. CTA+ is a Generally Regarded As Safe (GRAS) molecule that is already approved for use in personal care products and allows broad-spectrum antimicrobial action. We believe that the combination of these features contributes to overcoming the bottleneck of designing scalable antimicrobial surfaces for large-scale applications.

## Methods

### Preparation and Characterization of SAMs, Brushes, and Painted Surfaces

Detailed procedures for the synthesis of self-assembled monolayers, 3-sulfopropyl methacrylate brushes, and waterborne latex particles are reported in the Supporting Information. The thicknesses of the SAMs and brushes were determined via spectroscopic ellipsometry (SE-2000, Semiconductor Physics Laboratory Co., Ltd.). X-ray photoelectron spectroscopy (Kratos Axis Supra or PHI VersaProbe II scanning XPS) was performed to acquire high-resolution spectra of specific regions and confirm the loading of surfactants on the surface. Water contact angle (DataPhysics OCA 35) measurements were also performed.

Plastic coverslips were coated with the paint formulation by means of a brush. Care was taken to apply a thin layer of paint on the coverslip to avoid macro-cracking upon drying, which may cause peeling off of the coating. The paint was dried at room temperature for at least 1 day. The loading of CTA+ was performed either by dipping the coated surface in a 1 mM CTA+ aqueous solution for 30 min, by treating the surface with wipes prewetted in a 1 mM CTA+ solution, or by spraying a 1 or 10 mM CTA+ solution. Surfaces were washed 1, 7, 30, or 90 times with a microfiber cloth prewetted in Milli-Q water to evaluate the surface retention of CTA+ upon repeated washing or intentional surface contamination (see the Supporting Information for further details). The coating quality did not appear to be compromised after the dipping, spraying, or washing procedures.

### Antiviral Tests

The antiviral activity of the surfaces was evaluated by drying a virus inoculum on the surface and quantifying the residual virus titer by a plaque assay. In a general approach, surfaces (2 × 2 cm) were placed inside Petri dishes, and 80 μL of virus inoculum (∼10^5^ PFU/mL) was carefully spread over the surface. The inoculum was dried for 15–75 min on the surface and collected with a swab prewetted in the releasing media. The released virus titer was determined by cell infection, incubation, fixation (except for Pseudomonas phage phi6), and quantification of the number of plaque-forming units (PFU). Herpes Simplex Virus Type 2 (HSV-2, provided by M. Pistello, University of Pisa, Italy), Influenza virus A/Netherlands/602/2009 (H1N1, provided by Prof. M. Schmolke, University of Geneva, Switzerland), Pseudomonas phage phi6 (DSM 21518), and Severe Acute Respiratory Syndrome Coronavirus (SARS-CoV-2, B.1.1.7 variant, hCoV-19/Switzerland/un-2012212272, EPI_ISL_2131446, provided by Prof. I. Eckerle, University Hospital of Geneva, Switzerland) were tested on Vero (African green monkey fibroblastoid kidney cells, ATCC), MDCK (Madin-Darby Canine Kidney cells, ATCC), *Pseudomonas sp* (DSM 21482), and Vero E6 (ATCC) cells, respectively. Two biological duplicates were performed for each test (each involving technical duplicates). All the cells were cultured in Dulbecco’s modified Eagle’s medium (DMEM-GlutaMAX, Gibco/BRL, Gaithersburg, MD) supplemented with 10% heat-deactivated fetal bovine serum (FBS, Gibco/BRL, Gaithersburg, MD) and 1% penicillin/streptomycin (P/S, Gibco/BRL, Gaithersburg, MD) and grown in 5% CO_2_-humidified atmosphere at 37 °C. *Pseudomonas sp* (DSM 21482) was cultured at 30 °C under agitation in Tryptone soya broth (TSB) supplemented with 1% of CaCl_2_/Glucose.

### Antibacterial Tests

A similar protocol was followed to perform antibacterial tests on surfaces. Surfaces were placed inside Petri dishes, and 80 μL of an overnight liquid bacterial culture (adjusted to ∼10^5^–10^6^ CFU/mL) was carefully spread all over the surface to be tested. The inoculum was dried for 30 min up to 24 h depending on the microorganism. The residual bacteria on the surface were collected with a sterile swab prewetted with LB or 5-fold diluted Dey-Engley neutralizing broth in PBS at physiological pH (7.4) (NB). The colony-forming units were determined by growing colonies on agar plates. *Staphylococcus aureus* (ATCC 25923), *Escherichia coli* (ATCC 700728), and *Salmonella enteritidis* (ATCC BAA-1045) were obtained from ATCC. *Listeria monocytogenes* (L526, isolated from sandwich spread) was provided by Prof. M. Uyettendaele, University of Ghent, Belgium.

## Supporting Information Available

Dose–response assays of surfactants, characterization of SAMs (contact angle, thickness, XPS), antiviral tests on polymer brushes, characterization of the paint (SEM, contact angle, XPS), antiviral and antibacterial tests on painted surfaces; complete synthetic and characterization methods of SAMs, polymer brushes, and paint; and complete cell culture and antimicrobial tests methods (PDF)

## Abbreviations

ABS: acrylonitrile butadiene styrene
ASTM: American standard for testing materials
ATCC: American-type culture collection
BA: butyl acrylate
BSA: bovine serum albumin
CFU: colony forming units
CTA+: cetyltrimethylammonium ion
DMEM: Dulbecco’s modified Eagle’s medium
DS-: dodecylsulfate ion
FBS: fetal bovine serum
GRAS: generally regarded as safe
HR: high resolution
HSV-2: herpes simplex virus type-2
LB: liquid broth
MDCK: Madin-Darby Canine Kidney
MMA: methyl methacrylate
MUA: 11-mercaptoundecanoic acid
MUS: sodium 11-mercapto-1-undecanesulfonate
NB: neutralizing broth
OD: optical density
P/S: penicillin/streptomycin
PFU: plaque forming units
PP: polypropylene
PSPMA: polysulfopropyl methacrylate
QAC: quaternary ammonium compound
RB: recovered bacteria
RIV: recovered infectious virus
SAM: self-assembled monolayer
SARS CoV-2: severe acute respiratory syndrome coronavirus
SI-ATRP: surface-initiated atom transfer radical polymerization
TMA: 11-mercaptoundecyl-*N*,*N*,*N*-trimethylammonium bromide
XPS: X-ray photoelectron spectroscopy
