# Supplementary material for: Broad-Spectrum Supramolecularly Reloadable Antimicrobial Coatings
Source: ACS Appl Mater Interfaces. 2024 Jun 3;16(23):29867–75. doi: 10.1021/acsami.4c04705 (PMC11181266; doi:10.1021/acsami.4c04705)
Supplement: Supplementary file 1 — am4c04705_si_001.pdf [file am4c04705_si_001.pdf]

SUPPORTING INFORMATION

Broad-spectrum supramolecularly reloadable antimicrobial coatings

Fiora Artusio[1], *Lukas Müller*[1,2], *Nicolò Razza*[1], *Inês Cordeiro Filipe*[1], *Francesca Olgiati*[1], *Łukasz Richter*[1], *Edoardo Civera*[1], *Melis Özkan*[1], *Matteo Gasbarri*[1], *Louisa Rinaldi*[1], *Heyun Wang*[1], *Esther Garcìa*[3], *Julie Schafer*[3], *Lise Michot*[3], *Sophie Butot*[3], *Leen Baert*[3], *Sophie Zuber*[3], *Marcus Halik*[2], *Francesco Stellacci*[1,4*]

[1] Institute of Materials, Ecole Polytechnique Fédérale de Lausanne (EPFL), 1015 Lausanne, Switzerland

[2] Organic Materials & Devices, Institute of Polymer Materials, Friedrich-Alexander-Universsät Erlangen-Nürnberg, Interdisciplinary Center for Nanostructured Films (IZNF), Cauerstraße 3, 91058 Erlangen, Germany

[3] Nestlé Research, Institute of Food Safety and Analytical Sciences, Vers-chez-les-Blanc, Box 44, 1000 Lausanne, Switzerland

[4] Interfaculty Bioengineering Institute, Ecole Polytechnique Fédérale de Lausanne (EPFL), 1015 Lausanne, Switzerland.

* corresponding author: francesco.stellacci@epfl.ch

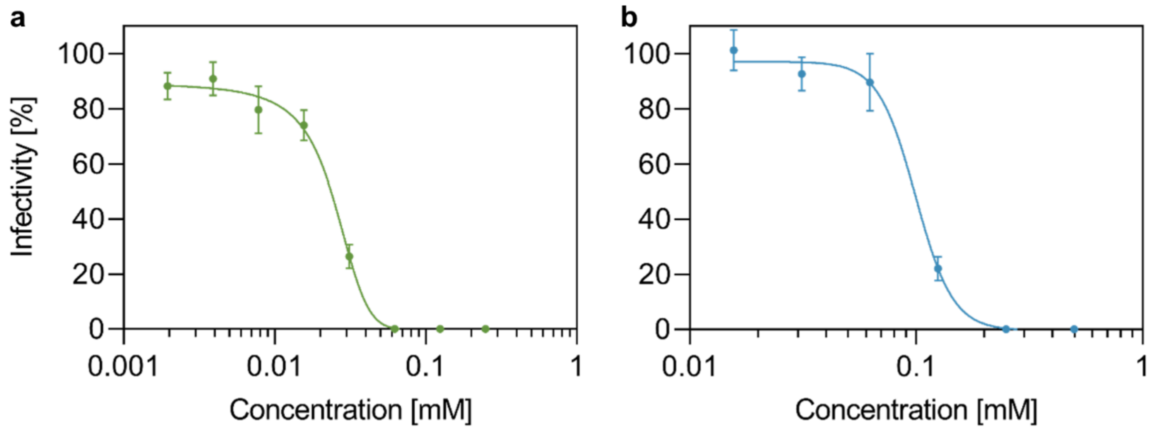

Figure S1. Dose-response assays of a) CTA+ and b) DS- against HSV-2. IC_{50} was 0.025 mM for CTA+ and 0.1 mM for DS-.

Table S1. Water contact angle and thickness as measured with ellipsometry of gold, as-prepared SAMs, and SAMs loaded with surfactants.

Surface	Water contact angle, °	Thickness, nm
**Gold**	60.7 (± 1.2)	-
**MUS-**	31.5 (± 0.9)	0.91 (± 0.04)
**MUS-/CTA+**	50.7 (± 1.0)	1.61 (± 0.08)
**MUA-**	56.1 (± 0.6)	0.93 (± 0.04)
**MUA-/CTA+**	65.9 (± 0.7)	1.64 (± 0.22)
**TMA+**	53.9 (± 2.0)	2.37 (± 0.23)
**TMA+/DS-**	66.7 (± 0.2)	2.63 (± 0.07)

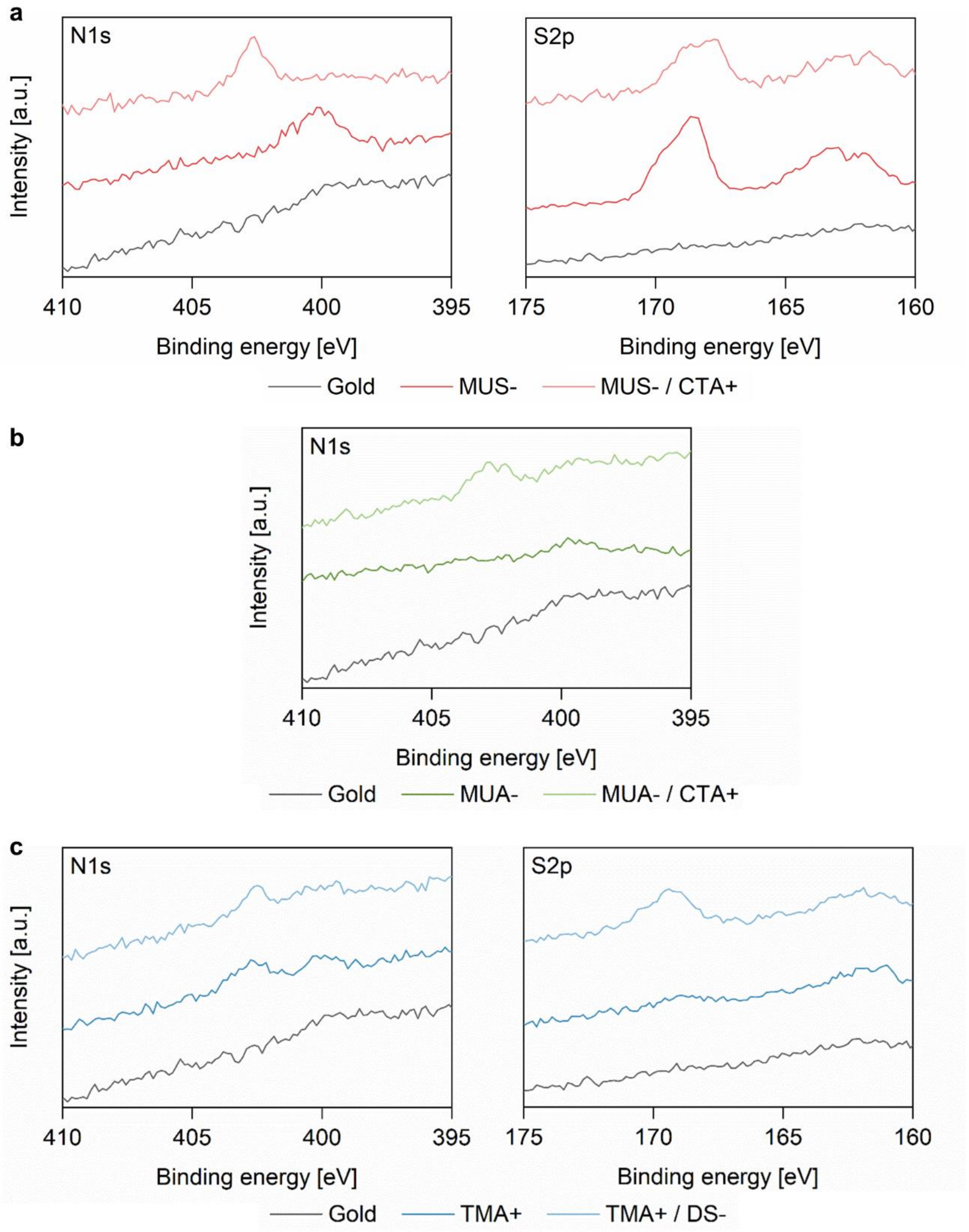

Figure S2. High resolution XPS spectra of a) N1s and S2p regions of gold, MUS- SAM and MUS- SAM loaded with CTA+, b) N1s region of gold, MUA- SAM and MUA- SAM loaded with CTA+ and c) N1s and S2p regions of gold, TMA+ SAM and TMA+ SAM loaded with DS-.

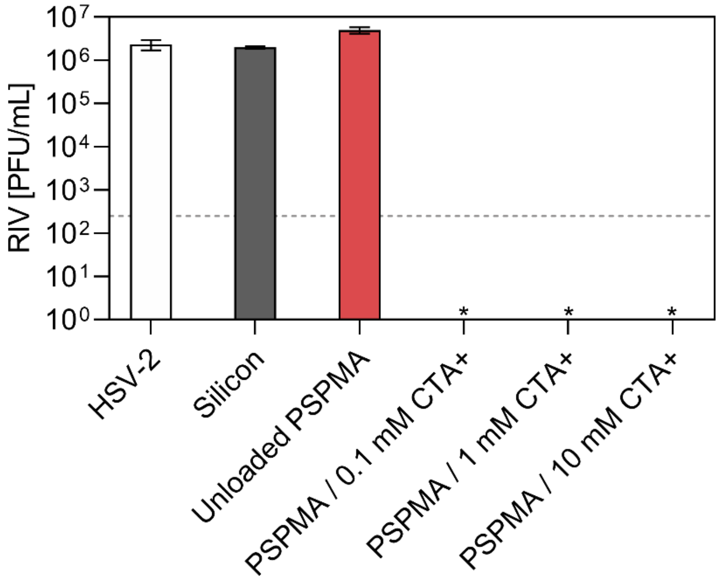

Figure S3. Antiviral test against HSV-2 (inoculum = 2×10^6 PFU/mL) performed on CTA+ loaded brushes at 120 min inoculation time and prevention of evaporation of the viral inoculum. The variation in loading concentration showed no effect. White bar refers to HSV-2 inoculum, grey bar refers to silicon controls, red bar to recovered virus on as-prepared PSPMA brushes. RIV stands for recovered infectious virus, as quantified by plaque assay. Dotted line corresponds to the limit of detection. The limit of detection was 250 PFU/mL. *stands for no observed infection.

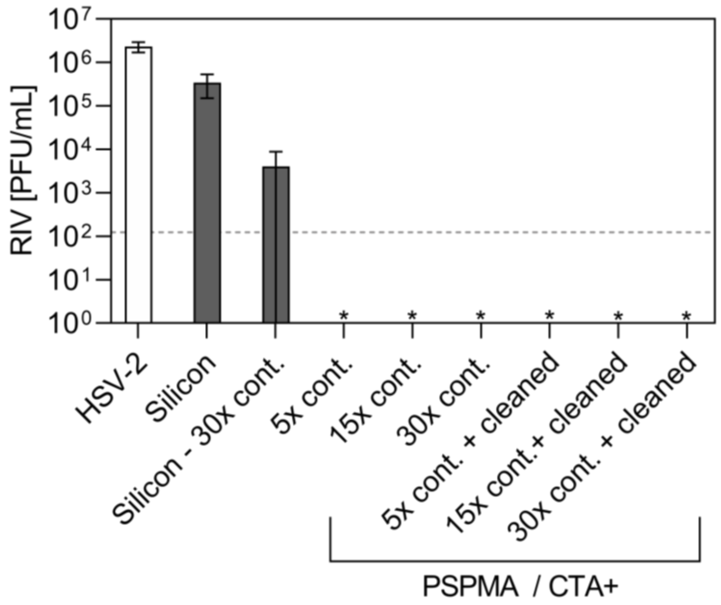

Figure S4. Antiviral tests against HSV-2 (inoculum = 2×10^6 PFU/mL, inoculation time = 75 min) performed on CTA+ loaded brushes after a) multiple contamination and cleaning cycles (5x, 15x, and 30x). "cont." stands for surface contamination with finger grease. White bar refers to HSV-2 inoculum, grey bars refer to silicon controls. RIV stands for recovered infectious virus, as quantified by plaque assay. Dotted line corresponds to the limit of detection. The limit of detection was 125 PFU/mL. * stands for no observed infection.

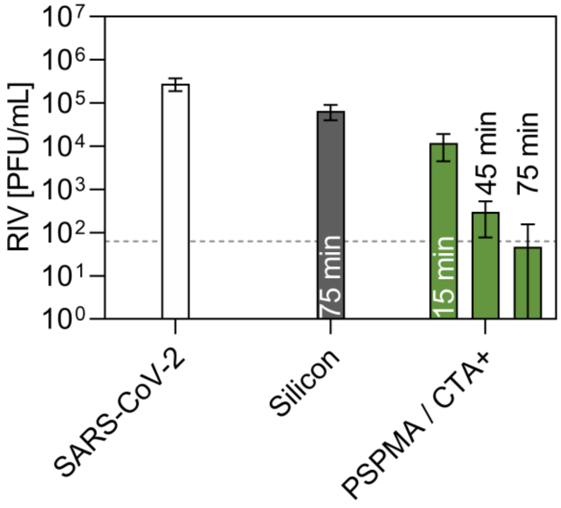

Figure S5. Antiviral test against SARS-CoV-2 (inoculum = 3×10^5 PFU/mL) performed on CTA+ loaded brushes at 15, 45, and 75 min inoculation time. White bar refers to SARS-CoV-2 inoculum, grey bar refers to silicon controls, green bars to recovered virus on CTA+ loaded PSPMA brushes. RIV stands for recovered infectious virus, as quantified by plaque assay. Dotted line corresponds to the limit of detection. The limit of detection was 62.5 PFU/mL.

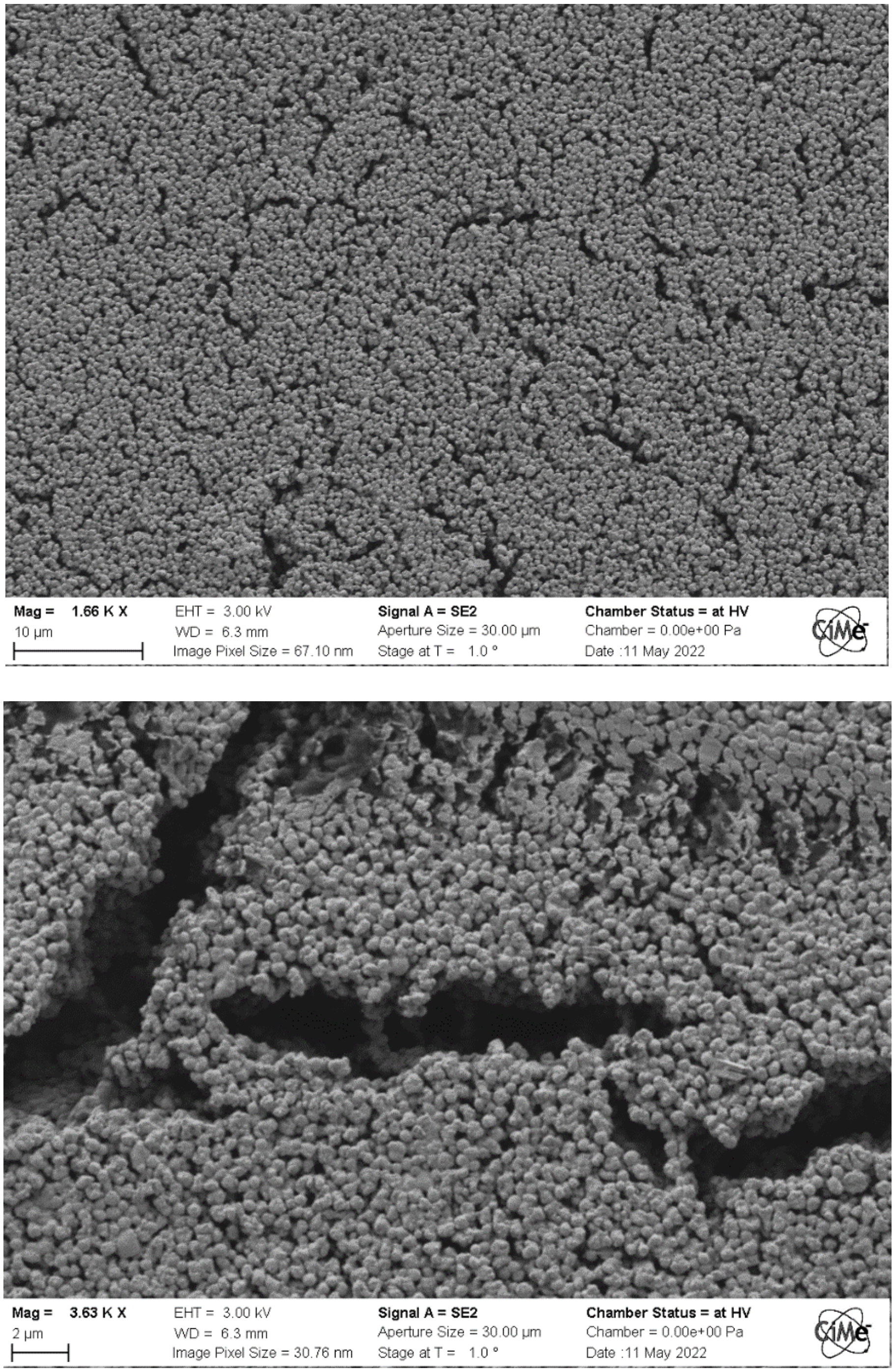

Figure S6. Micrographs of latex particles obtained by Scanning Electron Microscopy (SEM) at different magnifications (1.66K × and 3.63K ×).

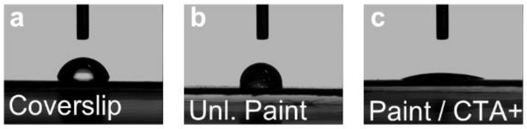

Figure S7. a) Water contact angle was 78.8 (± 0.4) º for the plastic coverslip, b) 97.2 (± 0.9) º for the unloaded paint, and c) 23.7 (± 1.9) º for the CTA+ loaded paint, suggesting a strong surface hydrophilicity increase due to the presence of surfactant.

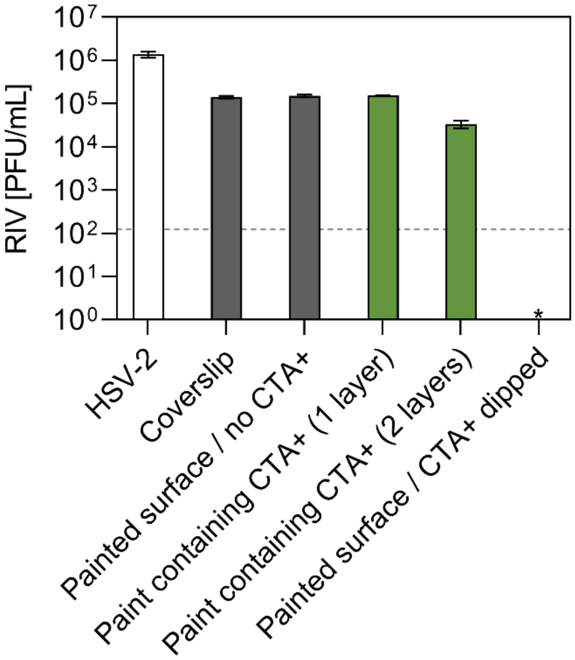

Figure S8. a) Antiviral test on HSV-2 (inoculum = 1×10^6 PFU/mL, inoculation time = 30 min) performed on paint formulations containing CTA+ and applied on the surface as a single (1 layer) or double (2 layers) layer, or on a paint formulation applied on the surface, dried, and dipped in CTA+ solution. White bar refers to HSV-2 inoculum, grey bars refer to controls (plastic coverslips and surfaces coated with the paint formulation but not loaded with CTA+), green bars to recovered virus on CTA+ loaded coating. RIV stands for recovered infectious virus, as quantified by plaque assay. Dotted line corresponds to the limit of detection. The limit of detection was 125 PFU/mL. *stands for no observed infection.

Table S2. Elemental composition (at%) of a painted coverslip loaded with CTA+ as obtained by XPS quantification.

C%	O%	N%	S%
69.2	25.9	3.1	1.8

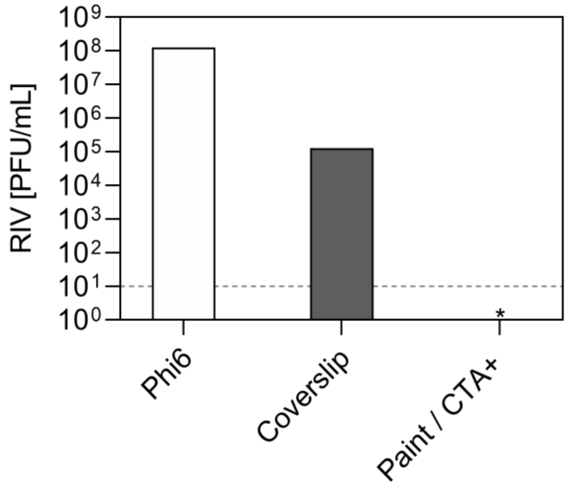

Figure S9. a) Antiviral test on Pseudomonas phage phi6 (inoculum = 1×10^8 PFU/mL, inoculation time = 2 h) performed on painted surfaces loaded with 1 mM CTA+. White bar refers to Pseudomonas phage phi6 inoculum, grey bar refers to plastic coverslip controls. RIV stands for recovered infectious virus, as quantified by plaque assay. Dotted line corresponds to the limit of detection. The limit of detection was 10 PFU/mL. *stands for no observed infection.

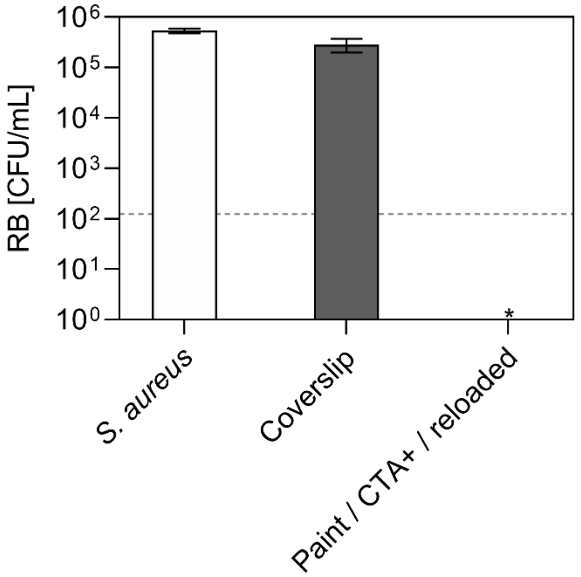

Figure S10. Antibacterial tests on *S. aureus* (inoculum = 5×10^5 CFU/mL, inoculation time = 30 min) performed on painted surfaces loaded with 1 mM CTA+ washed 90 times and reloaded by spraying 10 mM CTA+ solution. White bar refers to *S. aureus* inoculum, grey bar refers to plastic coverslip controls. RB stands for recovered bacteria. Dotted line corresponds to the limit of detection. The limit of detection was 125 CFU/mL. *stands for no observed infection.

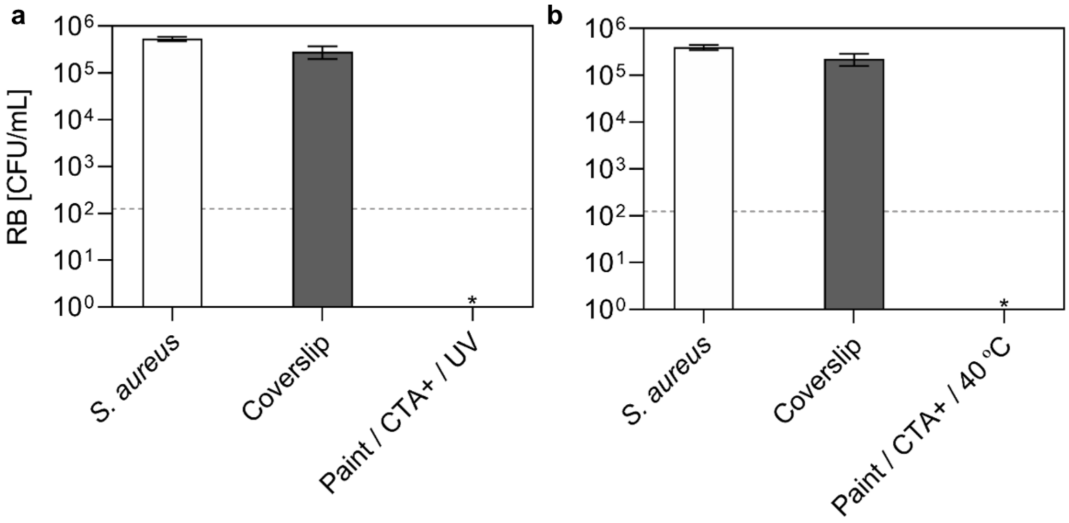

Figure S11. a) Antibacterial test on *S. aureus* (inoculum = 5×10^5 CFU/mL, inoculation time = 30 min) performed on coating exposed to UV light (250 W) for 4 hours. b) Antibacterial test on *S. aureus* (inoculum = 4×10^5 CFU/mL) performed on coating exposed to 40 ºC for 24 hours. White bars refer to *S. aureus* inoculum, black bars refer to plastic coverslip controls. RB stands for recovered bacteria. Dotted line corresponds to the limit of detection. The limits of detection was 125 CFU/mL. *stands for no observed infection.

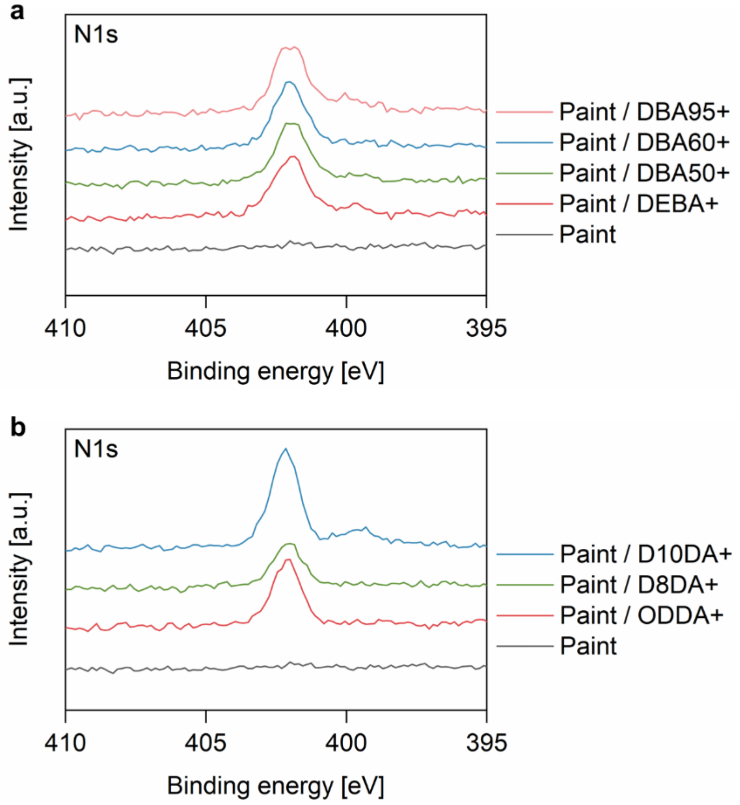

Figure S12. High resolution XPS spectra of N1s region of painted coverslips loaded with alternative QACs with a) one hydrocarbon chain and one aromatic ring and b) two hydrocarbon chains. DEBA+ stands for N-alkyl (68% C12, 32% C14) dimethyl ethylbenzyl ammonium, DBA50+ stands for N-alkyl (50% C14, 40% C12, 10% C16) dimethyl benzyl ammonium, DBA60+ stands for N-alkyl (60% C14, 30% C16, 5% C12, 5% C18) dimethyl benzyl ammonium, DBA95+ stands for N-alkyl (95% C14, 3% C12, 2% C16) dimethyl benzyl ammonium, ODDA+ stands for octyl decyl dimethyl ammonium, D8DA+ stands for dioctyl dimethyl ammonium, D10DA+ stands for didecyl dimethyl ammonium ions.

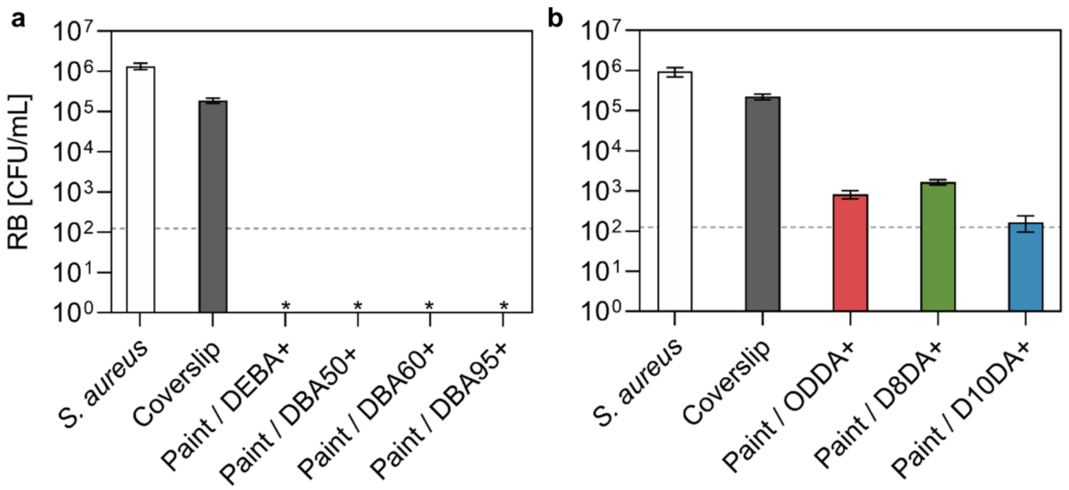

Figure S13. Antibacterial tests on *S. aureus* (inoculum = 1×10^6 CFU/mL, inoculation time = 30 min) performed on coating loaded with alternative QACs with a) one hydrocarbon chain and one aromatic ring and b) two hydrocarbon chains. DEBA+ stands for N-alkyl (68% C12, 32% C14) dimethyl ethylbenzyl ammonium, DBA50+ stands for N-alkyl (50% C14, 40% C12, 10% C16) dimethyl benzyl ammonium, DBA60+ stands for N-alkyl (60% C14, 30% C16, 5% C12, 5% C18) dimethyl benzyl ammonium, DBA95+ stands for N-alkyl (95% C14, 3% C12, 2% C16) dimethyl benzyl ammonium, ODDA+ stands for octyl decyl dimethyl ammonium, D8DA+ stands for dioctyl dimethyl ammonium, D10DA+ stands for didecyl dimethyl ammonium ions. RB stands for recovered bacteria. Dotted line corresponds to the limit of detection. The limit of detection was 125 CFU/mL. White bars refer to microorganism inoculums, and grey bars refer to surface controls. Red, green, and blue bars refer to recovered microorganisms from the painted surfaces loaded with different QACs. *stands for no observed infection.

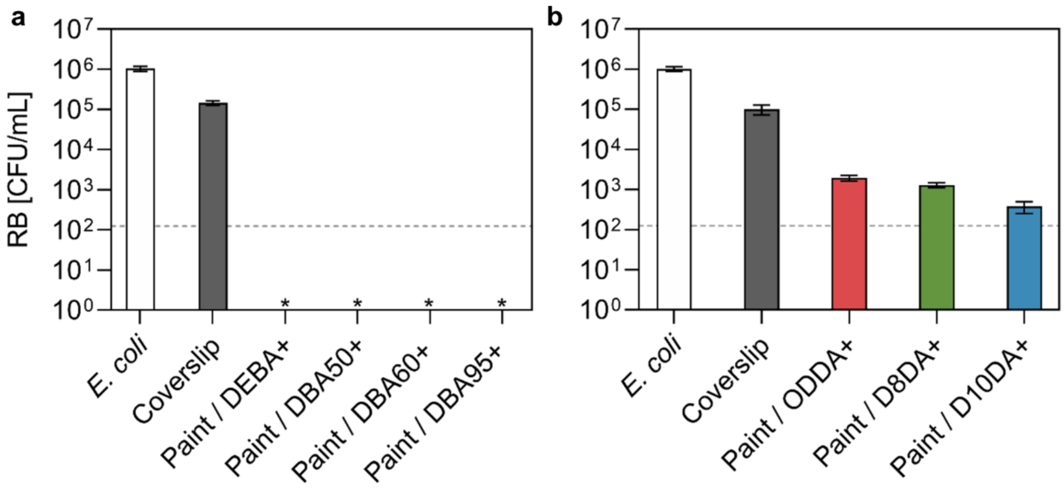

Figure S14. Antibacterial tests on *E. coli* (inoculum = 1×10^6 CFU/mL, inoculation time = 2 h) performed on coating loaded with alternative QACs with a) one hydrocarbon chain and one aromatic ring and b) two hydrocarbon chains. DEBA+ stands for N-alkyl (68% C12, 32% C14) dimethyl ethylbenzyl ammonium, DBA50+ stands for N-alkyl (50% C14, 40% C12, 10% C16) dimethyl benzyl ammonium, DBA60+ stands for N-alkyl (60% C14, 30% C16, 5% C12, 5% C18) dimethyl benzyl ammonium, DBA95+ stands for N-alkyl (95% C14, 3% C12, 2% C16) dimethyl benzyl ammonium, ODDA+ stands for octyl decyl dimethyl ammonium, D8DA+ stands for dioctyl dimethyl ammonium, D10DA+ stands for didecyl dimethyl ammonium ions. RB stands for recovered bacteria. Dotted line corresponds to the limit of detection. The limit of detection was 125 CFU/mL. White bars refer to microorganism inoculums, and grey bars refer to surface controls. Red, green, and blue bars refer to recovered microorganisms from the painted surfaces loaded with different QACs. *stands for no observed infection.

SUPPLEMENTARY MATERIALS AND METHODS

S1.1 Materials

Self-assembled monolayers (SAMs)

For the synthesis of SAMs, gold coated glass coverslips (22x22 mm, 50 nm gold thin film, Platypus Technologies, LLC) were used as substrates. Sodium 11-mercapto-1-undecanesulfonate (MUS) was synthesized according to the procedure described in literature[1]. 11-mercaptoundecanoic acid (95%, MUA), (11-mercaptoundecyl)-N,N,N-trimethylammonium bromide (TMA, ≥ 90 %), cetyltrimethyl ammonium bromide (CTAB, ≥ 99 %), and sodium dodecyl sulfate (SDS, ReagentPlus®, ≥98.5%) were purchased from Sigma-Aldrich.

Polymer brushes

For the synthesis of polymer brushes silicon wafer substrates (6”, 675 µm thick, boron doped, <100>) were obtained from Ted Pella, Inc. As initiator (3-trimethoxysilyl)propyl-2-bromo-2-methylpropionate was purchased from Gelest, Inc. Copper(II) bromide (99.999 %), copper(I) bromide (99.999 %), 2,2’-bipyridine (bpy, ≥ 99 %), 3-sulfopropyl methacrylate potassium salt (SPMA, 98 %), and ethylenediaminetetraacetic acid disodium salt dihydrate (EDTA-Na_2, for molecular biology) were purchased from Sigma-Aldrich. As common solvents acetone, methanol, and ethanol were used in HPLC grade only from multiple suppliers. Ultrapure water was obtained by treatment with a Milli-Q purification system.

Paint

For the synthesis of the paint, methyl methacrylate (MMA, 99%, contains ≤ 30 ppm MEHQ as inhibitor), butyl acrylate (BA, > 99%), sodium 4-vinylbenzene sulfonate (NaSS, > 90%), potassium persulfate (KPS, ≥ 98%), tert-butyl hydrogen peroxide (TBHP, Luperox, 70 wt% aqueous solution), hydrogen peroxide (HP, 30 wt% aqueous solution), ascorbic acid (AA, 99%) were purchased from Sigma-Aldrich. The paint was

loaded alternatively with cetyltrimethyl ammonium bromide (CTAB, Sigma-Aldrich), N-alkyl (68% C12, 32% C14) dimethyl ethylbenzyl ammonium chloride (DEBAC, Sigma-Aldrich), N-alkyl (60% C14, 30% C16, 5% C12, 5% C18) dimethyl benzyl ammonium chloride (DBAC60, FluoroChem), N-alkyl (95% C14, 3% C12, 2% C16) dimethyl benzyl ammonium chloride (DBAC95, Combi-Blocks), N-alkyl (50% C14, 40% C12, 10% C16) dimethyl benzyl ammonium chloride (DBAC50, Toronto Research Chemicals), octyl decyl dimethyl ammonium chloride (ODDAC, Toronto Research Chemicals), didecyl dimethyl ammonium chloride (D10DAC, Combi-Blocks), and dioctyl dimethyl ammonium chloride (D8DAC, LCG).

Cell culture

Vero (African green monkey fibroblastoid kidney cells, ATCC) cells, Vero E6 (ATCC) cells, and MDCK (Madin-Darby Canine Kidney cells, ATCC) cells were grown in 5% CO_2 humidified atmosphere at 37°C. All the cells were cultured in Dulbecco's modified Eagle's medium (DMEM-GlutaMAX, Gibco/BRL, Gaithersburg, MD) supplemented with 10% heat-deactivated fetal bovine serum (FBS, Gibco/BRL, Gaithersburg, MD) and 1% penicillin/streptomycin (P/S, Gibco/BRL, Gaithersburg, MD).

Viruses

Herpes Simplex Virus Type 2 (HSV-2) was provided by M. Pistello (University of Pisa, Italy). Influenza virus A/Netherlands/602/2009 (H1N1) was a kind gift from Prof. M. Schmolke (University of Geneva, Switzerland). Pseudomonas phage phi6 (DSM 21518) was obtained from DSM. Severe Acute Respiratory Syndrome Coronavirus (SARS-CoV-2) B.1.1.7 variant (hCoV-19/Switzerland/un-2012212272, EPI_ISL_2131446) was a generous gift from Prof. I. Eckerle (University Hospital of Geneva, Switzerland).

Bacteria

Staphylococcus aureus (ATCC 25923, STA121/125), *Escherichia coli* (ATCC 700728), and *Salmonella enteritidis* (ATCC BAA-1045) were obtained from ATCC. *Pseudomonas sp* (DSM 21482) was obtained from DSM. *Listeria monocytogenes* L526, isolated from sandwich spread, was a kind gift from Prof. M.

Uyettendaele, University of Ghent, Belgium. *Staphylococcus aureus*, *Escherichia coli*, and *Salmonella enteritidis* were cultured in sterile liquid broth (LB). *Pseudomonas sp* was cultured in tryptone soya broth (TSB) supplemented with 1 % of $CaCl_2$/Glucose.

S1.2 Synthesis of self-assembled monolayers on Au surfaces

Gold-coated coverslips were sonicated in acetone for 5 min, cleaned with ethanol and methanol, and blow-dried with argon. 1 mM thiol solutions (MUS, MUA, and TMA) were prepared in degassed methanol, sonicated for 15 min, and degassed for 5 min. Surfaces were placed inside glass vials and 20 mL of the thiol solution was added. The vials were closed, and the reaction proceeded for 24 h at 40 ºC. Surfaces were thoroughly rinsed with methanol and blow-dried with argon. For active compound-loaded surfaces, a dipping step followed. 0.1 mM solutions of CTAB or SDS were prepared in Milli-Q water and sonicated for 20 min. Surfaces were immersed in 20 mL solution for 30 min, thoroughly rinsed with Milli-Q water and dried with nitrogen.

S1.3 Synthesis of polymeric brushes

The synthesis of polymer brushes first involved the vapor phase deposition of the initiator[2]. Briefly, silicon wafers were cut into 2x2 cm^2 squares, rinsed, sonicated in acetone for 20 min, blow-dried, and plasma-cleaned (Diener electronic GmbH & Co KG) with oxygen plasma for 20 min. Immediately after the treatment, the substrates were placed in a desiccator containing 100 μL of (3-trimethoxysilyl)propyl-2-bromo-2-methylpropionate. The initiator deposition proceeded for 72 h under vacuum, followed by annealing at 60 ºC for 30 min in a vacuum oven. The initiator-coated substrates were stored under vacuum until further utilization.

Polymer brushes were synthesized via Surface-Initiated Atom Transfer Radical Polymerization (SI-ATRP) on the initiator-coated substrates[3]. A catalyst solution was prepared by adding 42.21 mg of copper(II) bromide and 324.85 mg of 2,2'-bipyridine (bpy) to a two-necked round-bottom Schlenk flask containing 15 mL of MilliQ water : methanol (4:1 vol:vol). The mixture was sonicated to achieve complete dissolution.

The solution was degassed to remove any oxygen with three cycles of freeze, pump, and thaw using a Schlenk line. After the third cycle, the solution was frozen again, and 119.49 mg of copper(I) bromide were added under argon. After thawing, the mixture was sonicated again to dissolve the copper(I) bromide leading to a color change from blue to dark red. A final freeze, pump, thaw cycle was performed. Meanwhile, a second flask containing 8.65 g of the monomer, 3-sulfopropyl methacrylate potassium salt (SPMA), was connected to the Schlenk line and flushed with argon for 2 h. The catalyst solution was then cannula-transferred to the SPMA-containing flask, followed by sonication. 1.5 mL of the solution were syringe-transferred to a previously evacuated glass vial containing the initiator-coated substrate. A typical synthesis was carried out for 40 min at room temperature (duration was varied for obtaining different polymer brush lengths), followed by reaction quenching with MilliQ water and methanol and blow-drying. To remove the Cu/bpy complex residues after the synthesis, PSPMA brushes were immersed in 20 mL of 10 mM EDTA-Na_2 in MilliQ water for 1 min. The substrates were thoroughly rinsed with MilliQ water and blow-dried with argon. As previously described for SAMs, PSPMA brushes were also loaded with cetyltrimethyl ammonium cation (CTA+) by immersion in 20 mL of 0.1 mM CTAB solution in MilliQ water for 30 min[4], followed by rinsing with MilliQ water and blow-drying.

S1.4 Synthesis of waterborne acrylic latex and preparation of the coating

The synthesis of the waterborne acrylic paint was inspired by Bilgin et al.[5]. Briefly, the synthesis was carried out in two steps. First, the seeds were prepared by adding 6.9 g of MMA, 6.9 g of BA, and 0.276 g of NaSS to 77 mL of Milli-Q water. The mixture was degassed with argon for 20 min. The reaction was carried out at 70 ºC under agitation upon addition of 3 mL of 0.13 M of KPS aqueous solution. After 3.5 h, the reaction was stopped, and the seeds were collected from the reactor. Then, the synthesis of the latex formulation was carried out in semi-continuous conditions. A four-necked round-bottom flask was loaded with 39.4 g of seeds. Three solutions were prepared to be fed separately to the reactor with the help of syringe-pumps. Feed 1 was made of 20.6 g of MMA and 20.6 g of BA; feed 2 was made of 0.462 g of NaSS, 0.111 g of AA, and 12.46 g of water; feed 3 consisted of 0.112 g of TBHP and 12.51 g of water. The three solutions

were fed over 20 min with feeding rate of 2 mL/min for feed 1 and 0.6 mL/min for feed 2 and 3. The reaction was allowed to proceed batchwise at 70 ºC under agitation for 2 h. Then, 0.96 g of HP were added as a shot and, after 1 min, 6.7 mL of a 0.4 M AA solution were added dropwise over 10 min. The reaction continued for 1 h and then the reactor was allowed to cool down to recover the latex formulation. The latex formulation was used to coat plastic coverslips with a brush. The coating was dried at room temperature for at least one day.

S1.5 Loading, washing, reloading of latex-coated surfaces

The loading of CTA+ was performed either by dipping the coated surface in 1 mM CTA+ aqueous solution for 30 min, by treating the surface with wipes pre-wetted in 1 mM CTA+ solution, or by spraying of 1 or 10 mM CTA+ solution. Then, surfaces were rinsed with Milli-Q water to remove any unbound CTA+ residue.

The painted surfaces loaded with CTA+ were washed and dried 1, 7, 30, or 90 times with a microfiber cloth pre-wetted in Milli-Q water to evaluate the surface retention of CTA+ upon repeated washing. When the antimicrobial activity was lost, surfaces were reloaded by dipping in 1 mM CTA+ solution for 30 min to restore the original antimicrobial properties.

S1.6 Ageing tests

To evaluate the performance of the coating under challenging environmental conditions, ageing tests were performed exposing the painted surfaces to UV light (250W, up to 4 hours) or heat (40 °C, up to 24 hours). The antimicrobial activity of the coating was then tested, as well as its optical appearance.

S1.7 Surface characterization

Water contact angle (WCA) measurements were performed using a DataPhysics OCA 35 contact angle measurement instrument in ambient conditions. A 2 μL MilliQ water droplet was used for each

measurement. The contact angle was determined using the software SCA20 from DataPhysics. The reported values are given as average value of at least 3 measurements ± standard deviation.

XPS measurements were carried out using a Kratos Axis Supra (Kratos) for SAMs and painted surfaces and a PHI VersaProbe II scanning XPS microprobe (Physical Instruments AG) for brushes. Survey scans and High-Resolution (HR) XPS spectra of N1s region were acquired. The analyses were performed using a monochromatic Al Kα X-ray source. The spherical capacitor analyzer was set at 45° take-off angle with respect to the sample surface. The spectra were calibrated setting the C1s hydrocarbon component at 284.8 eV.

Thicknesses of SAMs and brushes were determined via spectroscopic ellipsometry using an SE-2000 spectroscopic ellipsometer from SEMILAB (Semiconductor Physics Laboratory Co., Ltd.). Per substrate and condition three to nine individual spots were characterized. Each measurement consisted of an initial focusing step with a 633.93 nm light source at an incident angle of 70° followed by acquisition of reflected light at the same angle over a photon energy E_{ph} spectral range of 0.75 - 4.5 eV. A three-layer Gold-SAM-Air model and a five-layer Si-SiO_2-Initiator-Brush-Air model, of which the electromagnetic/optical properties and thicknesses of the underlying layers have been determined before, were established to describe the structure of the SAMs and the brushes, respectively. Cauchy and Lorentz models were selected for fitting the transmissive and absorptive properties, respectively.

Scanning Electron Microscopy (SEM) analyses were performed on latex particles. Silicon wafers (1x1 cm^2) were coated with a layer of latex and dried overnight under a fume hood. Samples were metallized with a 5-nm thick Au layer. SEM was operated at 3 kV and working distance was set at 6.3 mm.

S1.8 Antiviral tests

The antiviral activity of the surfaces was generally evaluated by drying a virus inoculum on the surface and quantifying the residual virus titer by plaque assay for HSV-2, Influenza virus, Pseudomonas phage phi6 and SARS-CoV-2. In a general approach, surfaces were placed inside Petri dishes, and 80 µL of virus inoculum (~10^5 PFU/mL for HSV-2, Influenza virus and Pseudomonas phage phi6, SARS-CoV-2) were

carefully spread over the surface. The inoculum was allowed to dry for 30 min ("wet-to-dry" protocol) and was then collected with a swab pre-wetted in the releasing media. Three viruses were tested. All the tests were performed in biological duplicates or triplicates. The methods used for quantification are reported below.

<u>HSV-2.</u> 1.45 x 10^4 Vero cells/well were plated in 96-well plates 24 h before the experiment. The cell medium was changed to DMEM/2%FBS/1%P/S before the infection. The swab was inserted into a vial containing 1000 µL DMEM-GlutaMAX /2%FBS/1%P/S and vortexed for 1 min. The released virus was used to infect the first row of the 96-well plate and then serial 3x dilutions were performed. After 1 h infection at 37°C, the cells were overlaid with medium containing 1.2% methylcellulose and incubated overnight at 37°C. On the following day, cells were stained with 0.1% of crystal violet in 20% ethanol.
As regards experiments conducted on brushes, the inoculum was dried on the surface at room temperature (following "wet-to-dry" protocol), and the remaining viral titer was collected after 15, 45, or 75 min with a cotton swab pre-wetted in 30 µL of 5-fold diluted Dey-Engley neutralizing broth in PBS at physiological pH (7.4) (NB). The swab was then transferred to a vial containing 970 µL of NB, and cell infection followed as previously described. Additional experiments were also carried out preventing the evaporation of the virus inoculum following a procedure adapted from ISO Standard 21702:2019[8] ("wet-to-wet" protocol). 80 µL of virus inoculum were spread over the surface and immediately covered with a coverslip. After 2 h, the coverslip was carefully removed. Both the surface and the coverslip were thoroughly washed with 1 mL of NB to collect the residual virus. Cell infection proceeded as previously described.

<u>Influenza virus.</u> 8.5 x 10^5 MDCK cells/well were plated in 6-well plates 24 h before performing the antiviral test. Before the infection, the cells were washed with PBS +/+ (with $MgCl_2$ and $CaCl_2$), and the medium was changed to DMEM /1%P/S. The cells were infected with 10-fold serial dilutions of the recovered viral inoculum prepared in DMEM-GlutaMAX/1%P/S at 37°C for 1 h. Then, the cells were overlaid. A 2X MEM stock was first supplemented with 4 mM L-Glutamine, 0.24% $NaHCO_3$, 0.02 M HEPES, 0.2 mg/mL

P/S, and 0.42% BSA, and then diluted 1:1 and supplemented with 0.6% agar, 1 µg/mL TPCK trypsin, 0.1 µg/mL DEAE dextran, and 1 mg/mL $NaHCO_3$. After 48 h of incubation at 37°C, the cells were fixed with 4% paraformaldehyde solution and stained with 0.5% crystal violet.

Pseudomonas phage phi6. Pseudomonas phage phi6 was propagated on *Pseudomonas sp* (DSM 21482) according to ISO 10705-1[7]. In short, *Pseudomonas sp* (DSM 21482) was cultured in conical flasks at 30°C under agitation in Tryptone soya broth (TSB) supplemented with 1% of $CaCl_2$/Glucose ($CaCl_2x2H_2O$ Sigma 223506, 3 g + Glucose Merck 1.08337, 10 g + 100 mL H_2O) to an approximate OD_{600} of 0.3 before adding Pseudomonas phage phi6 (10^7 PFU/mL) for an incubation of 16-18 h. Pseudomonas phage phi6 was recovered from the supernatant after centrifugation for 20 min at 3000 g and filtered through a 0.22 µm filter. For each experiment 80 µL of Pseudomonas phage phi6 inoculum (~10^5 PFU/mL) were dried on the surface at room temperature and collected after 2 h with a cotton swab pre-wetted in 30 µL of 5-fold diluted Dey-Engley neutralizing broth in PBS at physiological pH (7.4). Samples were serially diluted in Tryptone Salt (TS: NaCl Merck 1.06404, 8.5 g/L + Tryptone Oxoid LP0042, 1 g/L). Subsequently 100 µL of dilutions were put into contact with 100 µL of Pseudomonas sp (DSM 21482) cultured at 30°C under agitation in TSB supplemented with 1 % of $CaCl_2$/Glucose (OD_{600} of 0.3) for 15 minutes before adding 6 mL of TSB soft agar (TSB + Agar Oxoid LP0011 6 g/L) and pouring the mixture onto TSB agar plates (TSB + Agar Oxoid LP0011 20 g/L) which were incubated for 24 h ± 2 h at 30 °C.

SARS-CoV-2. 10^5 Vero E6 cells/well were plated in 24-well plates 24 h before the plaque assay. 3-fold/5-fold serial dilutions of the recovered viral inoculum were prepared in DMEM-GlutaMAX/2.5%FBS/1%P/S. Cell infection was performed at 37°C for 1 h. Then, cells we overlaid with DMEM-GlutaMAX/2.5%FBS/1%P/S containing 0.4% Avicel GP3515 and incubated at 37°C for 48 h. Cells were finally fixed with 4% PFA and stained with 0.1% crystal violet in 20% ethanol.

S1.9 Antibacterial tests

One day before the test, a liquid culture of bacteria was started. A colony of bacteria was withdrawn from an agar streak plate culture and transferred to liquid broth. The culture was incubated overnight at 37°C. The antibacterial activity of the surfaces was tested by letting the bacteria inoculum to evaporate on the surface. Surfaces were placed inside Petri dishes, and 80 µL of the overnight liquid bacterial culture adjusted to ~10^5-10^6 CFU/mL) were carefully spread all over the surface to be tested. The inoculum was allowed to dry for 30 min – 24 hours depending on the test without closing the Petri dish. The residual bacteria on the surface were collected with a sterile swab pre-wetted with LB or NB. The swab was inserted into a vial containing 1 mL of LB or NB. 10x serial dilutions were performed and 100 µL of inoculum were spread on an agar plate with the help of a cell spreader. The plates were allowed to dry for 15 min and incubated overnight at 37°C in static conditions. On the next day, the number of colonies in each plate was evaluated. Each surface was analyzed in triplicates.

S1.10 Contamination tests

To simulate real-life application scenarios, the antimicrobial properties were also tested in the presence of organic contamination. A preliminary study on polymer brush coated substrates (2x2 cm) was carried out by introducing intentional surface contamination via fingertips touching. One surface contamination was defined by four touching events by four different fingers of a participant's hand to transfer as much finger grease as possible on the whole surface area of the substrate. It was made sure that the hands have not been previously moisturized or polluted in some way. Polymer brush coated substrates were contaminated 5, 15 and 30 times by multiple volunteers. Samples were produced in quadruplicates. To mimic a typical cleaning scenario, two samples of each contamination count were additionally sprayed once with a 10 mM aqueous CTA+ solution in Milli-Q water and wiped once with a microfiber cloth to dry the surface. The reference uncoated silicon substrates (duplicates) were contaminated 30 times. The virucidal effect against HSV-2 (75 min of inoculation on surface) was evaluated not later than 24 h after the contamination to avoid aging of the grease.

In the following part of the study, the soil contamination was standardized according to ASTM E2197[6]. 0.05 g/mL bovine serum albumin (BSA), 0.05 g/mL tryptone, and 0.004 g/mL bovine mucin stock solutions were prepared in PBS. The contaminated virus/bacteria inoculum was prepared adding 50 μL of BSA solution, 70 μL of tryptone solution, and 200 μL of mucin solution to 680 μL of virus/bacteria stock. The concentration of the uncontaminated inoculum was adjusted by adding 320 μL of PBS to 680 μL of virus/bacteria stock. Alternatively, for surface contamination experiments, 50 μL of BSA solution, 70 μL of tryptone solution, and 200 μL of mucin solution were added to 680 μL of PBS. Then, 80 μL of soil mixture were deposited on the surface and dried at room temperature. Then, the general protocol for testing the antiviral/antibacterial properties of the surface followed.

REFERENCES

(1) Cagno, V.; Andreozzi, P.; D'Alicarnasso, M.; Jacob Silva, P.; Mueller, M.; Galloux, M.; Le Goffic, R.; Jones, S. T.; Vallino, M.; Hodek, J.; Weber, J.; Sen, S.; Janeček, E.-R.; Bekdemir, A.; Sanavio, B.; Martinelli, C.; Donalisio, M.; Rameix Welti, M.-A.; Eleouet, J.-F.; Han, Y.; Kaiser, L.; Vukovic, L.; Tapparel, C.; Král, P.; Krol, S.; Lembo, D.; Stellacci, F. Broad-Spectrum Non-Toxic Antiviral Nanoparticles with a Virucidal Inhibition Mechanism. *Nat. Mater.* **2018**, 17 (2), 195–203. https://doi.org/10.1038/nmat5053.

(2) Dunlop, I. E.; Thomas, R. K.; Titmus, S.; Osborne, V.; Edmondson, S.; Huck, W. T. S.; Klein, J. Structure and Collapse of a Surface-Grown Strong Polyelectrolyte Brush on Sapphire. *Langmuir* **2012**, 28 (6), 3187–3193. https://doi.org/10.1021/la204655h.

(3) Rzhepishevska, O.; Hakobyan, S.; Ruhal, R.; Gautrot, J.; Barbero, D.; Ramstedt, M. The Surface Charge of Anti-Bacterial Coatings Alters Motility and Biofilm Architecture. *Biomater. Sci.* **2013**, 1 (6), 589. https://doi.org/10.1039/c3bm00197k.

(4) Zhang, R.; Ma, S.; Wei, Q.; Ye, Q.; Yu, B.; van der Gucht, J.; Zhou, F. The Weak Interaction of Surfactants with Polymer Brushes and Its Impact on Lubricating Behavior. *Macromolecules* **2015**, 48 (17), 6186–6196. https://doi.org/10.1021/acs.macromol.5b01267.

(5) Bilgin, S.; Bahraeian, S.; Liew, M. L.; Tomovska, R.; Asua, J. M. Surfactant-Free Latexes as Binders in Paint Applications. *Prog. Org. Coat.* **2022**, 162, 106591. https://doi.org/10.1016/j.porgcoat.2021.106591.

(6) E35 Committee. *Quantitative Disk Carrier Test Method for Determining Bactericidal, Virucidal, Fungicidal, Mycobactericidal, and Sporicidal Activities of Chemicals*; ASTM International. https://doi.org/10.1520/E2197-17E01.

(7) International Organization for Standardization. *Water Quality-Detection and Enumeration of Bacteriophages-Part 1: Enumeration of F-specific RNA Bacteriophages*. ISO, 10705-1:1995.

(8) International Organization for Standardization. *Measurement of antiviral activity on plastics and other non-porous surfaces*. ISO, 21702:2019.
